# Investigating Breast Cancer Cell Behavior Using Tissue Engineering Scaffolds

**DOI:** 10.1371/journal.pone.0118724

**Published:** 2015-04-02

**Authors:** Khadidiatou Guiro, Shyam A. Patel, Steven J. Greco, Pranela Rameshwar, Treena L. Arinzeh

**Affiliations:** 1 Department of Biomedical Engineering, New Jersey Institute of Technology, Newark, New Jersey, United States of America; 2 Department of Medicine, Rutgers New Jersey Medical School, Newark, New Jersey, United States of America; Universita' degli Studi del Salento, ITALY

## Abstract

Despite early detection through the use of mammograms and aggressive intervention, breast cancer (BC) remains a clinical dilemma. BC can resurge after >10 years of remission. Studies indicate that BC cells (BCCs) with self-renewal and chemoresistance could be involved in dormancy. The majority of studies use *in vitro*, two-dimensional (2-D) monolayer cultures, which do not recapitulate the *in vivo* microenvironment. Thus, to determine the effect of three-dimensional (3-D) microenvironment on BCCs, this study fabricated tissue engineering scaffolds made of poly (ε-caprolactone) (PCL) having aligned or random fibers. Random and aligned fibers mimic, respectively, the random and highly organized collagen fibers found in the tumor extracellular matrix. Chemoresistant BCCs were obtained by treating with carboplatin. Western blot analysis of carboplatin resistant (treated) MDA-MB-231 (highly invasive, basal-like) and T47D (low-invasive, luminal) BCCs showed an increase in Bcl-2, Oct-4 and Sox-2, suggesting protection from apoptosis and increase in stem-like markers. Further studies with MDA-MB-231 BCCs seeded on the scaffolds showed little to no change in cell number over time for non-treated BCCs whereas on tissue culture polystyrene (TCP), non-treated BCCs displayed a significant increase in cell number at days 4 and 7 as compared to day 1 (p<0.05). Treated BCCs did not proliferate on TCP and the fibrous scaffolds. Little to no cyclin D1 was expressed for non-treated BCCs on TCP. On fibrous scaffolds, non-treated BCCs stained for cyclin D1 during the 7-day culture period. Treated BCCs expressed cyclin D1 on TCP and fibrous scaffolds during the 7-day culture period. Proliferation, viability and cell cycle analysis indicated that this 3-D culture prompted the aggressive BCCs to adopt a dormant phenotype, while the treated BCCs retained their phenotype. The findings indicate that random and aligned fibrous PCL scaffolds may provide a useful system to study how the 3-D microenvironment affects the behavior of BCCs.

## Introduction

In the United States, breast cancer is the most prevalent cancer and the second most common cause of cancer death among women. It is estimated that one in nine women will develop breast cancer in her lifetime and about one-third of whom will succumb to the disease [1] [2]. Despite improvement of early diagnosis and treatment, breast cancer remains a clinical problem [3]. In the absence of surgery, ionizing radiation and chemotherapeutic agents are the frontline therapies for the local control of breast cancer. However, with these non-surgical treatments, the principal issue becomes the lack of specificity for cancer cells alone, thus the cytotoxic effects on normal healthy cells limit both therapies. Moreover, recent studies have reported that another issue concerning radiation and chemotherapeutic agents is that cancer stem (tumor-initiating) cells remain dormant and acquire resistance to these conventional therapies [4–7]. Breast cancer recurrence or metastatic reactivation may be a result of these stem cells remaining dormant. It is well known that dormant tumor cells can stay in a quiescent state for many years as single cells that are resistant to therapies that target proliferating cells [8, 9]. In 2003, Al-Hajj et al. was the first group to describe breast cancer stem cells (BCSCs) to be a minority (0.1–5%) of the tumor but possessing the capability of limitless proliferation. BCSCs are also described to have the capacity for long-term self-renewal, to transition to a dormant phenotype and resist existing therapeutic agents such as carboplatin, and initiate distant metastatic disease [10, 11]. Iovino et al. recognized that these cells have low rates of cell division, exhibit resistance to primary chemotherapy and radiation and are characterized by surface expression of CD44+ (hyaluronan receptor) and CD24- (P-selectin) [4, 12]. Moreover, BCSCs can express anti-apoptotic proteins, MDR proteins, and retain efficient DNA repair mechanisms [4, 13–15]. Specifically, an immature subset of BCCs (Oct4^hi^/CD44^hi/med^/CD24^−/+^) has been identified that demonstrates chemoresistance, dormancy, and stem cell properties of self-renewal, serial passaging ability, cycling quiescence, long doubling time, asymmetric division, high metastatic and invasive capability [4]. Several studies are targeting these cells to eliminate their self-renewal capabilities. However, a better understanding of the mechanism of dormancy is needed to isolate, identify and treat these cells [5, 11, 15–19] [20, 21] [22–33].

Studies focus on the significance of the microenvironment in the progression, metastasis and dormancy of breast cancer. Within the stromal extracellular matrix (ECM), type I collagen has been shown to be one of the major components, which plays an important role during normal breast tissue development [34]. Notably, high breast tissue density due to increased collagen [35] is one of the single largest risk factors for developing breast cancer [36]. Furthermore, tumor cells have been shown to localize near randomly oriented dense collagen fibers, which can lead to tumor growth and expansion of the collagen matrix leading to matrix reorganization [37, 38] and alignment allowing facilitated local invasion [37, 39, 40]. Currently, the cell-based two-dimensional (2-D) monolayer cultures used as *in vitro* models present several limitations that three-dimensional (3-D) tissue engineering scaffolds/models can address [41–44]. For instance, studies by Bissell et al. described that suitable 3-D cultures could provide a more physiologically relevant approach to the analysis of gene function and cell phenotype ex vivo [45] while cancer cells cultured in 2-D poorly represented their *in vivo* physiological conditions. Moreover, the ability of malignant cells to grow and metastasize *in vivo* depends upon specific cell-cell and cell-extracellular matrix (ECM) interactions, many of which are absent when cells are cultured on conventional 2-D tissue culture plastic [46]. Consequently, when engineering the tumor microenvironment, scaffolds that allow for effective cell seeding and penetration, and have controllable architectural features and material properties are desirable [47]. Fibers are the basic structural elements of the ECM, thus engineered nonwoven fibrous scaffolds have found application in the field of tissue engineering where they are widely used to investigate and promote cell growth and tissue formation for a wide variety of cells and tissue types [48–51]. A commonly used processing method called electrospinning allows for the formation of fibrous scaffolds having nano- and microscale fiber diameters with either random or aligned fiber arrangements [52, 53]. Although many studies have reported growth of normal tissues on electrospun scaffolds, little is known about the behavior of tumor cells on these fibers [54]. Therefore, the purpose of this study was to investigate breast cancer cells (BCCs), specifically examining the subset of chemoresistant BCCs, on electrospun scaffolds as an effective *in vitro* model to study breast cancer cell behavior.

In this study, 3-D scaffolds, fabricated using electrospinning, consisted of micron-sized random and aligned fibers to mimic the orientation and size of collagen fibers in the native ECM. As mentioned by Provenzano et al., the ECM surrounding the normal mammary epithelial and tumor cells is comprised of collagen fibrils (~67 nm) which bundle to form micron-sized collagen fibers [37]. Breast cancer cells can orient along aligned fibers, but the significance of this fiber arrangement for tumor cell behavior is still not well understood. The fibers were made of poly (ε-caprolactone) (PCL), which is a biocompatible polymer with a low glass transition temperature and favorable mechanical properties at room and body temperatures. It is commonly investigated for tissue engineering scaffolds and is a slowly degrading material and therefore, a suitable composition for an *in vitro* model [55]. The electrospun scaffold was characterized for morphology, fiber diameter, inter-fiber spacing, porosity, and mechanical properties. BCCs were investigated in the 3-D *in vitro* environment and comparisons were made with 2-D monolayer cultures. Chemo-resistant BCCs were identified by cell viability and characterized for stem cell markers. Both chemoresistant (treated) and non-treated BCCs were evaluated on the scaffolds, specifically for attachment, growth, cell cycle analysis, apoptosis proteins and stem cell markers with the goal of identifying the effect of the 3-D environment on these populations.

## Materials and Methods

### Reagents

Dulbecco’s Modified Essential Medium (DMEM), penicillin, streptomyosin, Alexa Fluor 488 phalloidin and RNase A were purchased from Invitrogen (Carlsbad, CA). Roswell Park Memorial Institute (RPMI) 1640 was purchased from Sigma (St. Louis, MO). Restore Western Blot Stripping Buffer and NE-PER Nuclear and Cytoplasmic Extraction Kit were purchased from Thermo Scientific (Waltham, MA), fetal bovine serum from Hyclone (Logan, UT), restore western blot stripping buffer and NE-PER Nuclear and Cytoplasmic Extraction Kit from Thermo Scientific (Waltham, MA). Poly (ε -caprolactone) (PCL, [(CH2)5COO]n-), having 80,000 MW, was purchased from Sigma Aldrich, Inc. The solvent used for electrospinning was methylene chloride (density = 1.32g/cm^3^, boiling point = 39.75°C, dielectric constant = 9.1).

### Antibodies

The following antibodies were purchased from Abcam (Cambridge, MA): rabbit polyclonal anti-Oct4, anti-bax rabbit monoclonal, rabbit polyclonal anti-Sox2, rabbit polyclonal to Cyclin D1, mouse monoclonal IgG to β-actin, mouse anti-β-actin mAb, anti-Bcl-2 antibody, FITC- polyclonal goat anti-rabbit IgG. Rabbit anti-goat IgG—Rhodamine conjugate from Millipore (Billerica, MA); 4'-6′-diamidino-2-phenylindole nuclear stain (DAPI) and green phalloidin were purchased from Invitrogen (Carlsbad, CA). Anti-human CD44-PE, and anti-human CD24-FITC were purchased from BD Biosciences (Franklin Lakes, NJ). PE-anti-rabbit IgG was purchased from Santa Cruz Biotechnology (Santa Cruz, CA).

### Cell Lines

MDA-MB-231 (highly invasive, basal-like) and T47D (low-invasive, luminal) breast cancer cell lines were obtained from the American (ATCC). The cells were propagated as per ATCC instructions. Briefly, MDA-MB-231 were cultured in DMEM containing 10% fetal bovine serum and 1% penicillin and streptomyosin, at 37°C, 5% CO_2_ in a humidified incubator. T47D breast cancer cell lines were cultured in RPMI 1640 supplemented with 10% FBS and 0.2 Units/ml bovine insulin. Media was replaced every 2–3 days. The cells were split at 80% confluence using 0.25% EDTA-trypsin.

### Scaffold Fabrication Using Electrospinning

PCL scaffolds were electrospun using previously described methods[56]. Briefly, 15wt% PCL was dissolved in methylene chloride. The electrospinning setup used for the fabrication of scaffolds consists of a syringe pump (Cole Parmer, Vernon Hills, IL), a syringe containing a polymer solution, a needle attached to the syringe, a grounded collector (aluminum plate) and a high-voltage power supply (Gamma high voltage, Florida). A 10-ml plastic disposable syringe, 20-gauge needle, 20 kV voltage, infusion rate of 3 ml/h and distance of 40 cm between the syringe needle and grounded collector, were the parameters used for the fabrication of the electrospun mats. For aligned fiber electrospinning, instead of a collector plate, a rotating drum was used for aligned fiber collection. The drum rotated at 1000 to1300 rpm during the process. The electrospinning parameters to collect aligned fibrous scaffolds from the drum were similar to the random fiber collection. Scaffold thicknesses were 0.36 ± 0.02 mm for random fibers and 0.30 ± 0.03 mm for aligned fibers. The electrospun mats were air dried for 1 day to remove any residual solvents and stored in vacuum desiccators.

### Scaffold Characterization

The average fiber diameter, inter-fiber spacing, degree of fiber alignment for aligned fibrous scaffolds and porosity for each scaffold was determined using scanning electron microscopy (SEM, LEO 1530 Gemini, Germany) following previously published work. Briefly, three samples from each mat were evaluated using five different regions for each sample. SEM images were taken and a total of 15 fiber-diameters and 15 inter-fiber spacings for each mat were calculated using Image J software (National Institutes of Health, USA). Degree of fiber alignment was determined by forming a line perpendicular to a fiber in each 1000X image using Image J. Mechanical properties were determined by tensile testing using an Instron 3342 mechanical tester (Instron Corporation, Norwood, MA, USA) using previously reported methods [57]. The porosity was determined by the difference between the density of the electrospun scaffold and the unprocessed material, as previously reported [56]. The porosity was calculated using the formula: Porosity (%) = (1-D_mat_/D_raw_) *100, where D_mat_ was determined by dividing the mass of the scaffold by its volume. Draw is the density of the raw material, PCL, which is 1.14 g/cm^3^ as given by the manufacturer. Thirty samples were measured for each scaffold to find the porosity.

### Carboplatin Treatment of BCCs

BCSCs have been shown to resist chemotherapy thus to isolate these CSCs from the heterogeneous non-treated population of BCCs, carboplatin sensitivity of different BCCs was investigated. MDA-MB-231 and T47D cells were seeded in T-175 tissue culture flasks. At 80% confluency, the growth media was replaced with fresh media containing different concentrations of carboplatin. At days 2 and 3-post treatment, the total number of non-viable cells was determined by trypan blue exclusion. In order to demonstrate chemo-resistant (treated) BCCs remain viable even with additional carboplatin treatment, a survival curve was created using different amounts of carboplatin. BCCs were pre-treated with 600 μg/ml of 5-Fluorouracil (5-FU) + 50 μg/ml Carboplatin for 3 days. The concentrations of drugs that were finally toxic to at least 50% of the cell population (IC_50_) were determined. Then, studies were performed looking at viability over the full range of carboplatin concentrations and compared to non-treated BCCs. Viability was assayed at one day using trypan blue exclusion

### Western Analyses

Carboplatin cell death can occur through an apoptotic pathway, and inhibition of this pathway by genes such as Bcl- 2 can lead to drug resistance. In human breast cancer cells, Bcl-2 and Bax, the inhibitor of Bcl-2, are constitutively expressed to tightly regulate apoptosis [58, 59]. One of many factors leading to breast malignancy is the up-regulation of bcl-2 gene expression, bax down-regulation ultimately resulting in the inhibition of apoptosis [60]. Alongside being chemoresistant, these cells can express genes linked to pluripotency. Therefore, western blot was performed to detect Bax, Bcl- 2, Oct-4 and Sox-2 for the different BCC cell lines treated with increasing concentrations of carboplatin (0, 55, 110, 220 μg/ml). Briefly western blot was performed as previously described [4]. For intracellular proteins, whole cell extracts were prepared with the NP-40 buffer and nuclear/cytoplasmic extracts with NE-PER Nuclear and Cytoplasmic Extraction kit. For membrane proteins, extracts were prepared with Qproteome Plasma Membrane Protein kit (Qiagen). BCC extracts (20 μg) were subjected to electrophoresis on 4–20% SDS-PAGE (Bio-Rad; Hercules, CA). Proteins were transferred to PVDF membranes, and membranes were incubated overnight in the respective primary antibodies. This was followed by 2 h incubation with HRP- conjugated secondary antibodies at 1:2000 final dilutions. The latter was detected with chemiluminescence. Membranes were stripped with Restore Western Blot Stripping Buffer and then re-probed for other proteins, includingβ-actin mAb (1:4000 dilution). All bands were normalized to β-actin.

### CD44/CD24 Expression

Our previous work examined tumorsphere potential of the chemoresistant BCC [4] and determined after serial passaging, the chemoresistant BCCs (Oct4 high) were 96% efficient at forming tumorspheres whereas the Oct4 low (progenitors) failed to undergo serial passage. We studied a similar population of cells in this study. The chemoresistant/treated BCCs, post 3 days of carboplatin treatment (220 μg/ml), were examined for cell surface markers CD44/CD24 by flow cytometry. CD44/CD24 is used to identify BCSC in human cell lines. These biomarkers are associated with increased resistance to chemotherapy, tumorgenesis, and poor prognosis. For cell surface labeling, treated BCCs were washed with PBS, fixed in 4% formaldehyde, and incubated with anti-CD44-PE for 30 min followed by a second labeling with anti-CD24-FITC. All incubations occurred for 30 min at 37°C in the dark. Cells were immediately analyzed by gating cells based on specific target emission with the FACSCalibur (BD Biosciences, San Jose, CA). The data was analyzed with CellQuest software (BD Biosciences, San Jose, CA). BCCs were analyzed based on size with Forward-scattered light (FSC) and Side-scattered light (SSC), then we further analyzed the fluorescence properties of this mixed population to identify particular cells expressing the surface markers CD44/CD24. Control MDA-MB-231 and T47D isotypes were also analyzed.

### BCCs on Scaffolds

From the western results and carboplatin survival curves obtained for the different BCCs cell lines, MDA-MB-231 cells were selected to be treated with 200 μg/ml of carboplatin and cultured on the scaffolds. For all experiments, the electrospun scaffolds with a thickness of 0.12 ± 0.02 mm were cut into 6 mm diameter discs following previously published work [56]. The samples were sterilized by immersing in 100% ethanol for 25 minutes and air-dried under sterile conditions over night. The scaffolds were then transferred to 96 well polypropylene non-adherent tissue culture plates (BD Biosciences, San Jose, CA) for cell seeding. Carboplatin treated MDA-MB-231 and non-treated MDA-MB-231 were seeded onto scaffolds at 31 cells/mm^2^ to obtain single cells on the scaffolds. The seeded BCCs were analyzed for attachment, proliferation, viability cyclin D1 expression and cell cycling phases. Comparisons were made with cells cultured on standard tissue culture treated polystyrene plates (TCP). The medium was changed at each time point and treated cells were supplemented with fresh medium containing carboplatin.

### Cell Morphology

At 1, 4 and 7 days, BCCs on scaffolds were evaluated for cell morphology. Cells on the scaffolds were washed with 1X PBS, fixed with 4% formaldehyde for 20 min and then permeabilized with 0.1% Triton X-100 in PBS. The cells were labeled with DAPI for nuclear identification and Alexa Fluor 488 phalloidin for actin filaments. After 1, 4 and 7 days, the cells were assessed for morphology by confocal microscopy (Clsi, Nikon, Japan) and SEM.

### Cell Proliferation

Cell proliferation was assessed on days 1, 4, and 7. The lysates were used to quantify cell number using the PicoGreen dsDNA assay (Invitrogen Corp., Carlsbad, CA) in which cell number can be correlated to fluorescence intensity. PicoGreen dsDNA reagent is an ultrasensitive fluorescent nucleic acid stain for quantifying double-stranded DNA in solution. BCCs of known cell number served as standards. Standards and samples (n = 4 per group per time-point) were prepared by lysing cells with 0.1% Triton X-100. Fluorescence was detected with a microplate reader (FLX800, Biotek, Winooski, Vermont) at 480 nm excitation/520 nm emission.

### Cell Viability

The metabolic activity of the cells was determined on days 1, 4, and 7. Viability was assessed in cell cultures using the CellTiter-Glo Cell Viability Assay (Promega) as per manufacturer’s protocol.

### Immunocytochemistry

The expression of Cyclin D-1 (CD1), Bax, Bcl2 and Oct4 in BCCs plated on the scaffolds was performed at days 1, 4, and 7 by confocal microscopy. Samples were washed with 1X PBS, fixed with 4% formaldehyde (10 min) and then permeabilized by incubating in 1% BSA / 10% normal goat serum / 0.3 M glycine in 0.1% PBS-Tween for 1h. The serum blocked the interaction between the antibody and non-specific proteins. The cells were then incubated overnight at 4°C with the anti-cyclin D1 (1/1000), anti-Bax (1/100), anti-Bcl2 (1/500), anti-Oct4 (1/100), and anti-Sox2 (1/300). The cells were incubated for 1 h at room temperature with a secondary (red) rabbit anti-goat IgG—Rhodamine conjugate at1/250 dilution. The cells were counterstained for nuclear identification (blue) with DAPI, and F-actin using green phalloidin.

### Cell Cycle Analyses

Cell cycle analyses cells were performed with 1 x 10^4^ BCCs. Samples were washed with 1X PBS and cells were detached from scaffolds using 0.25% EDTA-trypsin and neutralized with standard growth media. Cells were washed in PBS, fixed with 3.7% formaldehyde for 20 min and then resuspended in 0.1% hypotonic sodium citrate solution containing 50 μg/ml propidium iodide and 200 μg/ml DNase-free RNase A. Cells were incubated for 30 min at room temperature and then immediately analyzed on FACSCalibur (BD, San Jose, CA). Flow cytometric analyses data were analyzed with BD CellQuest software. After obtaining the results for cell growth/viability on the scaffolds, only the non-treated BCCs were evaluated for cell cycle analysis.

### Statistical Analyses

All assays were performed with an n = 4 per group per time point for each data point. Studies were also repeated to establish reproducibility of the data. Results in graphs are presented as mean ± standard deviation. Results were analyzed using a one-way ANOVA and a posthoc Tukey test. Statistically significant values were defined for p < 0.05. All statistical analyses were performed using SPSS Statistics software.

## Results

### Scaffold Characterization

**Fig 1:**
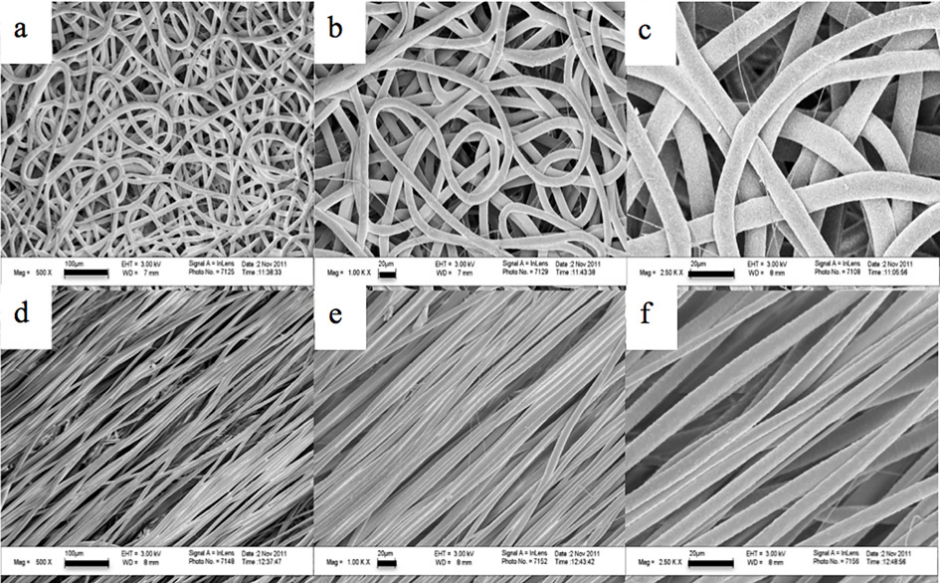
SEM micrographs of fibrous scaffolds. a–c) 500X, scale bar = 100 μm, 1000X, scale bar = 20 μm, and 2500X, scale bar = 20 μm magnifications, respectively, for random fibrous scaffolds. d–f) 500X, scale bar = 100 μm, 1000X, scale bar = 20 μm, and 2500X, scale bar = 20 μm magnifications, respectively, for aligned fibrous scaffolds.

Random and aligned fibrous scaffolds were produced as shown in Fig. 1. The fibers were uniform in morphology. PCL random fiber scaffolds had an average fiber diameter of 9.5 ± 2.2 μm, interfiber spacing of 86.2 ± 16.4 μm, elastic modulus of 4.4 ± 1.0 MPa and an ultimate tensile stress of 1.1 ± 0.1 MPa. PCL aligned fiber scaffolds had average fiber diameters of 8.9 ± 2.1 μm, interfiber spacing of 8.4 ± 1.7 μm and 95.4 ± 4.8% degree of alignment, an elastic modulus of 5.1 ± 0.8 MPa and an ultimate tensile stress of 0.8 ± 0.1 MPa. No significant differences were detected in fiber diameters and mechanical properties between the random and aligned fibers. Significant differences were detected for interfiber spacing between random and aligned fibers (p<0.05). Porosity was 85.2 ± 1.5 for random fibers and 76.8 ± 2.9 for aligned fibers, which were also significantly different (p<0.05).

### Carboplatin Survival Curves

**Fig 2:**
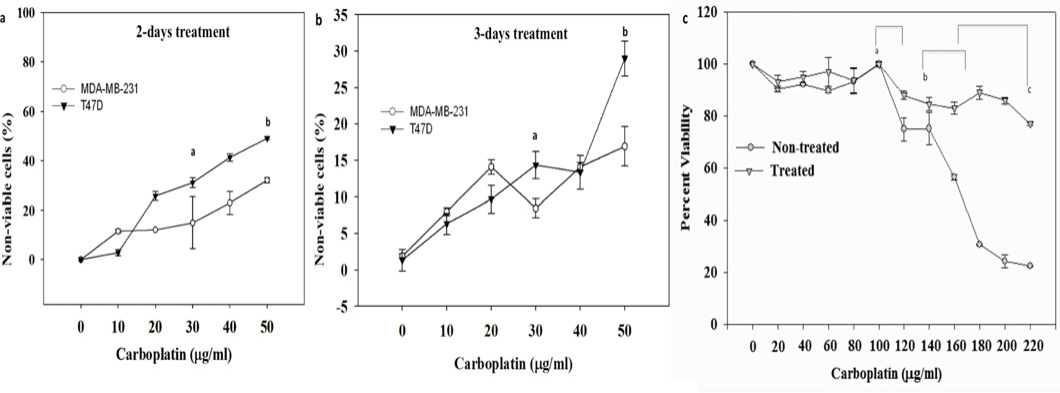
Effect of carboplatin treatment on the viability of breast cancer cell lines. a) Percentage of non-viable cells at 2 days post treatment with carboplatin. The results are shown as the mean±SD, n = 7 of non-viable cells. ^a^ p<0.05, significant increase in non-viable BCCs at 30 μg/ml as compared to 0 μg/ml. ^b^ p<0.05, significant increase in non-viable BCCs at 50 μg/ml as compared to 0 μg/ml and 30 μg/ml. b) Percentage of non-viable cells 3 days post treatment. ^a^ p<0.05, significant increase in non-viable BCCs at 30 μg/ml as compared to 0 μg/ml. ^b^ p<0.05, significant increase in non-viable BCCs at 50 μg/ml as compared to 0 μg/ml and 30 μg/ml. c) Carboplatin survival curve for chemotherapy treated and non-treated MDA-MB-231 cells, ^a^ p<0.05, significant decrease in percent viability (30%) of non-treated cells treated with carboplatin dosages between 100 μg/ml to 120 μg/ml as compared to treated cells (10%). ^b^ p<0.05, significant decrease in percent viability (40%) of non-treated cells treated with carboplatin dosages between 140 μg/ml to 170 μg/ml as compared to treated cells (20%).^c^ p<0.05, significant decrease in percent viability (80%) of non-treated cells treated with carboplatin dosages 170 μg/ml to 220 μg/ml as compared to treated cells (20%).

Carboplatin cytotoxicity was assessed after exposure to concentrations ranging from 0 to 50 μg/ml for 72 hours. At 2 days post treatment, all cell lines displayed sensitivity to carboplatin: 28% death (IC_50_ = 105.38 μM) for T47D cells and 17% death for MDA-MB-231 cells (IC_50_ = 182.1μM) at a maximum carboplatin concentration of 50 μg/ml (Fig. 2A). At 3 days post treatment, the maximum dosage of 50 μg/ml carboplatin concentration showed an increased sensitivity in T47D cells with 48% death (IC_50_ = 48.9 μM) (Fig. 2B). MDA-MB-231 cells had only 25% death (IC_50_ = 86 μM) 3 days post treatment. Furthermore, in order to demonstrate chemo-resistant (treated) BCCs remain viable even with additional carboplatin treatment, different concentrations of carboplatin were used on both non-treated and treated BCCs. Comparisons between non-treated and treated MDA-MB-231 showed no significant difference in percent viability between 0 and 100 μg/ml carboplatin treatment. However, with higher carboplatin dosages, non-treated cells showed a significant decrease in percent viability (p<0.05): 30% decrease between 100 μg/ml to 120 μg/ml carboplatin treatment as compared to treated cells (10%), 40% decrease between 140 μg/ml to 170 μg/ml carboplatin treatment as compared to treated cells (20%); 80% decrease between 170 μg/ml to 220 μg/ml carboplatin treatment as compared to treated cells (20%). Thus, treated BCCs had a percent viability of 80% plateauing at a high concentration of 220 μg/ml; unlike non-treated BCCs, which had a significant decrease in percent viability (20%) at this same concentration (p<0.05). (Fig. 2C).

### Western Analyses

**Fig 3:**
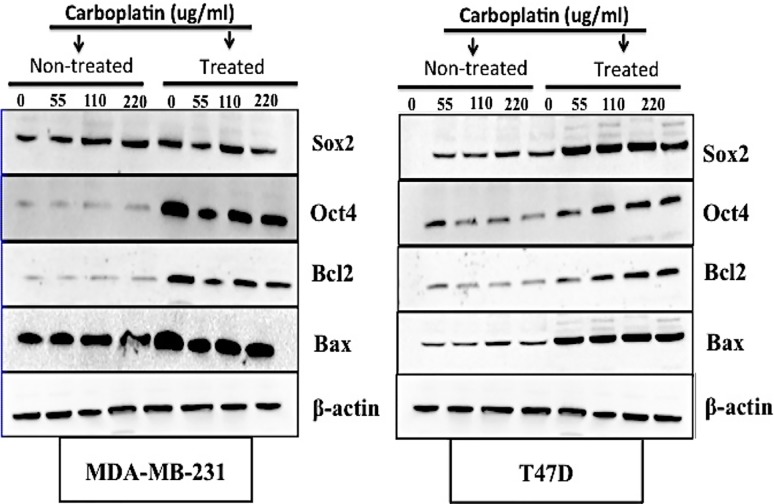
Western blot of breast cancer cell lines with chemotherapy treatment. Bax, Bcl2, Oct4, and Sox2 expression was determined for A) MDA-MB-231 cells and B) T47D cells. Densitometric bands normalized to β-actin have been provided in supplemental S1 Fig.

Western blots with antibodies that detected Bax, Bcl2, Oct4 and Sox2 proteins were performed and the results indicated predicted bands at 21, 30, 45 and 40 kDa respectively for MDA-MB-231 and T47D cells (Fig. 3A and Fig. 3B). The band intensities were higher for Bcl2 (S1A.b and f Fig.) and Oct4 (S1B.c and g Fig.) for treated BCCs cell types (MDA-MB231 and T47D cells) as compared to the non-treated BCCs cell types. Band intensities were higher for Sox2 (S1B.d and h Fig.) for treated T47D cells.

### CD44/CD24 Expression

**Fig 4:**
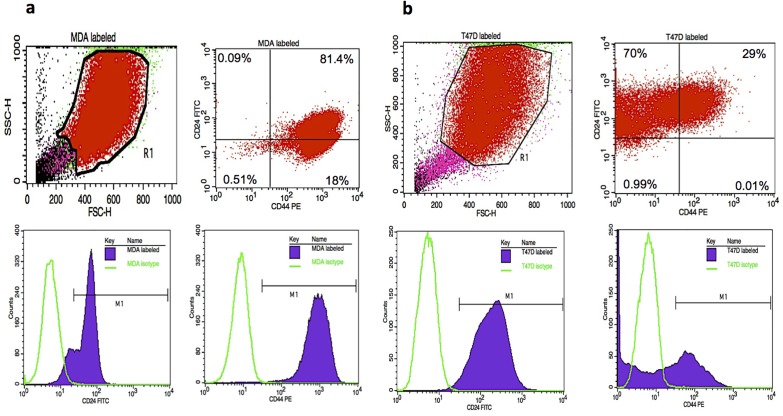
Flow cytometry analysis of CD44/CD24 expression of A) Top left: suspension of MDA-MB-231 treated cells analyzed based on size with forward-scattered light (FSC) and side-scattered light (SSC). Top right: R1 population further analyzed by specifically gating for CD44/CD24 expressing cells. Bottom left: histogram of MDA isotype control and CD24-FITC with histogram marker M1 designating CD24-FITC positive events. Bottom right: histogram of MDA isotype control and CD44-PE with histogram marker M1, designating CD44-PE positive events. B) Top left: suspension of T47D treated cells analyzed based on size with FSC and SSC. Top right: R1 population further analyzed by specifically gating for CD44/CD24 expressing cells. Bottom left: histogram of T47D isotype control and CD24-FITC with histogram marker M1 designating CD24-FITC positive events. Bottom right: histogram of T47D isotype control and CD44-PE with histogram marker M1, designating CD44-PE positive events.

The flow cytometry results showed that within the MDA-MB-231 treated cells: 0.09% were CD44^-^/CD24^+^; 81.4% were CD44^+^/CD24^+^; 0.51% were CD44^-^/CD24^-^ and 18% were CD44^+^/CD24^-^ (Fig. 4A top right). Treated T47D cells were: 70% CD44^-/low^/CD24^+^; 29% CD44^+^/CD24^+^; 0.99% CD44^-^/CD24^-^; 0.01% CD44^+^/CD24^-^ (Fig. 4B top right). Histograms of MDA isotype control with CD24-FITC (Fig. 4A bottom left) and CD44-PE (Fig. 4A bottom right) depicted CD24-FITC positive events and CD44-PE positive events. Similarly, histograms of T47D isotype control with CD24-FITC (Fig. 4B bottom left) and CD44-PE (Fig. 4b bottom right) showed CD24-FITC positive events and CD44-PE positive events.

### BCCs Interactions with 3-D Scaffolds

**Fig 5:**
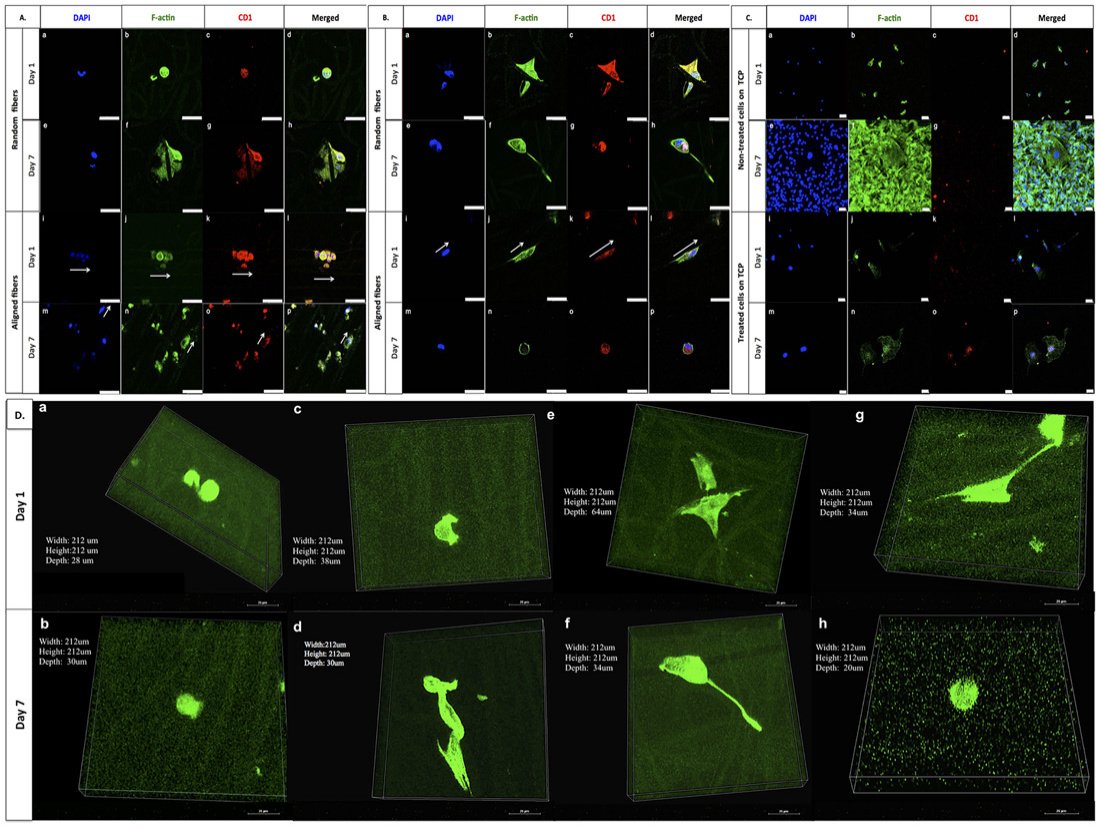
Confocal fluorescent microscope images of MDA-MB-231 BCCs on the PCL random and aligned fibrous scaffolds and TCP control. Blue indicates nuclei (DAPI); green indicates F-actin (Alexa 488) and red is for anti-cyclin D1 expression. A) Non-treated BCCs on random scaffolds (a through d at day 1; e through h at day 7) and aligned scaffolds (i through l at day 1; m through p at day 7). B) Treated BCCs on random scaffolds (a through d at day 1; e through h at day 7) and aligned scaffolds (i through l at day 1; m through p at day 7). C) Non-treated BCCs (a through d at day 1; e through h at day 7) and treated BCCs (i through l at day 1; m through p at day 7) on TCP. All scale bars are 50 μm. D) Volume View of MDA-MB-231 BCCs, green indicates F-actin. On random fibers, non-treated cells at a) day 1 and b) day 7, and treated cells at e) day 1 and f) day 7. On aligned fibers, non-treated cells at c) day 1 and d) day 7 and treated cells at g) day 1 and h) day 7. 60x objective. Scale bar is 25 μm. The arrows show the cell body orientation along the fibers.

Morphology and interaction between cells and scaffold fibers were analyzed *in vitro* for 7 days. Both carboplatin-resistant (treated) and non-treated MDA-MB-231 cells were adherent to the scaffolds. The non-treated cells had a round morphology at day 1 on both random and aligned fibers and at day 7, the non-treated cells appeared to be more spread or elongated on both fiber configurations (Fig. 5A). For treated cells at day 1, their morphology was spread on aligned fibers, with cell bodies along the fibers (Fig. 5B) but varied for cells on random fibers where some cells also appeared rounded (Fig. 5B and S3 Fig.). By day 7, treated cells appeared to have a more rounded morphology on random fibers but the morphology varied on aligned fibers where some cells appeared elongated along the fibers (S3 Fig.). On TCP, non-treated cells displayed confluency by day 7 with some cells characterized with spread and spindle-like shapes. For treated cells on TCP, cells appeared to be well-attached to the substrate with a spread morphology (Fig. 5C). Moreover the volume views of cells in the 3-D-reconstructed Z-stack images showed that both non-treated and treated MDA-MB-231 BCCs were present within the fibrous scaffolds (Fig. 5D). SEM imaging at higher magnification also was performed to examine adhesion and morphology of the MDA-MB-231 cells after 7 days on random and aligned fibrous scaffolds.

**Fig 6:**
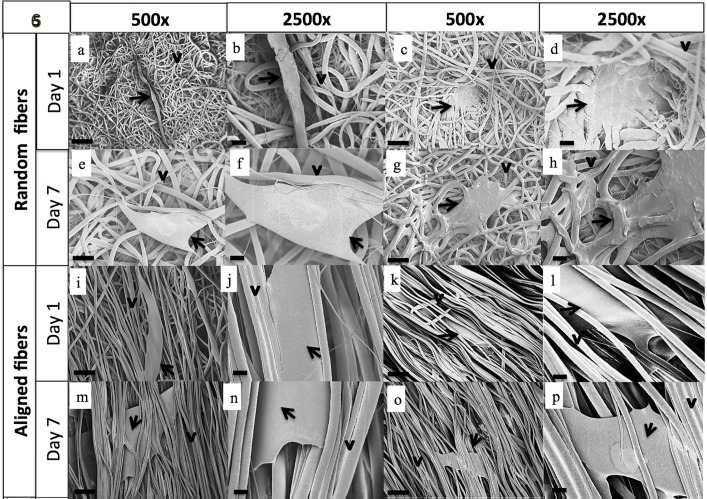
SEM images of MDA-MB-231 cells on fibrous scaffolds after day 1 and day 7 of culture. The arrows depict the cell body and the arrowheads depict the fibers. Supplemental data showing higher magnification analysis of adhesion and infiltration has been provided in S2 Fig.

On random fibrous scaffolds, non-treated cells were found on the top surface of the fibers at day 1 (Fig. 6A and 6B) and by day 7 they had larger, more spread cell bodies (Fig. 6E and 6F) interacting with the fibrous scaffolds. On aligned fibrous scaffolds, non-treated BCCs aligned their cell bodies in the direction of the fibers as early as day 1 (Fig. 6I and 6J) and were found on the top surface of the fibrous scaffolds. By day 7, non-treated BCCs on aligned fibers, displayed larger cell bodies and were within the aligned fibers (Fig. 6M and 6N). Treated/chemoresistant cells on random fibers displayed a more rounded cell shape at day 1 (Fig. 6C and 6D) and larger cell body by day 7 (Fig. 6G and 6H). On aligned fibers, treated cells aligned their cell bodies along the fibers at day 1 (Fig. 6K and 6L) and had large cell bodies by day 7. Cells on both random and aligned fibrous scaffolds displayed cell-cell (S2C, D Fig.) and cell-fiber (S2A-C Fig.) interactions. SEM demonstrated that cancer cells were attached to the scaffold. Individual cancer cell and cancer cell aggregates attached and spread on the surface of the fibrous scaffold and many cells attached to neighboring cells as shown (S2C Fig.).

### Cell Proliferation

**Fig 7:**
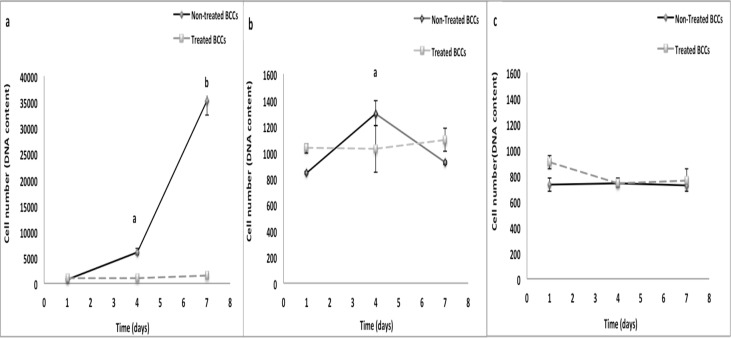
A: BCC growth on random and aligned fibrous scaffolds in comparison to TCP. a) TCP. ^a^ p<0.05, significant increase in growth of non treated BCCs at day 4 as compared to day 1. ^b^ p<0.05, significant increase in growth of non-treated BCCs at day 7 as compared to day 1and day 4. b) Random fibers. ^a^ p<0.05, significant increase in growth of non-treated BCCs at day 4 as compared to day 1 and day 7. c) Aligned fibers. Values are mean ±SD.

On TCP, non-treated BCCs displayed significant increase in cell number at days 4 and 7 as compared to day 1 (p<0.05) (Fig. 7A). Non-treated BCCs showed a slight increase in cell growth at day 4 (p<0.05) on random fibrous scaffolds (Fig. 7B). No differences in cell number were detected over time for treated BCCs on TCP and fibrous scaffolds. (Fig. 7A-C).

### Cell Viability

**Fig 8:**
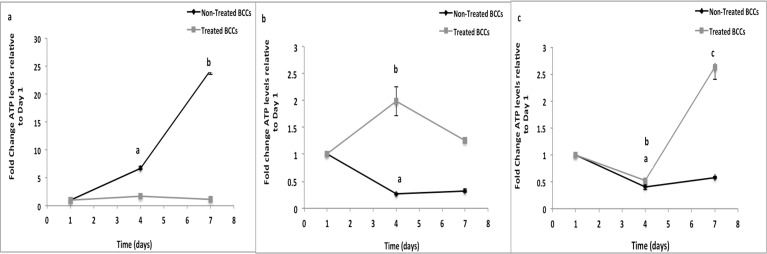
Metabolic activity of BCCs on random and aligned fibrous scaffolds in comparison to TCP. a) TCP. ^a,b^ p<0.05, significant increase in metabolically active non-treated BCCs at day 4 and day 7 as compared to day 1. b) Random fibers. ^a^ p<0.05, significant decrease in metabolic activity of non-treated BCCs as compared to day 1. ^b^ p<0.05, significant increase in metabolic activity of treated BCCs at day 4 as compared to day 1. c) Aligned fibers. ^a^ p<0.05, significant decrease in metabolic activity of non-treated BCCs as compared to day 1. ^b^ p<0.05, significant decrease in metabolic activity of treated BCCs as compared to day 1. ^c^ p<0.05, significant increase in metabolic activity of treated BCCs in comparison to days 1 and 4. Values are mean ±SD.

On TCP, non-treated BCCs displayed a significant increase in metabolic activity (p<0.05) over time (~7 fold and ~25 fold increase as compared to day 1) (Fig. 8A). Non-treated BCCs had significantly lower metabolic activity during the 7-day culture period on fibrous scaffolds as compared to TCP (Fig. 8A-C). On TCP, no differences in metabolic activity were detected for treated BCCs over the 7-day culture period. On random fibrous scaffolds, treated BCCs had a 2-fold (p<0.05) significant increase in metabolic activity at day 4 as compared to day 1 (Fig. 8B). Treated BCCs on aligned fibrous scaffolds showed a significant decrease (p<0.05) in metabolic activity at day 4 and a significant increase in metabolic activity (p<0.05) at day 7 (Fig. 8C).

### Cell Cycle Analyses

**Fig 9:**
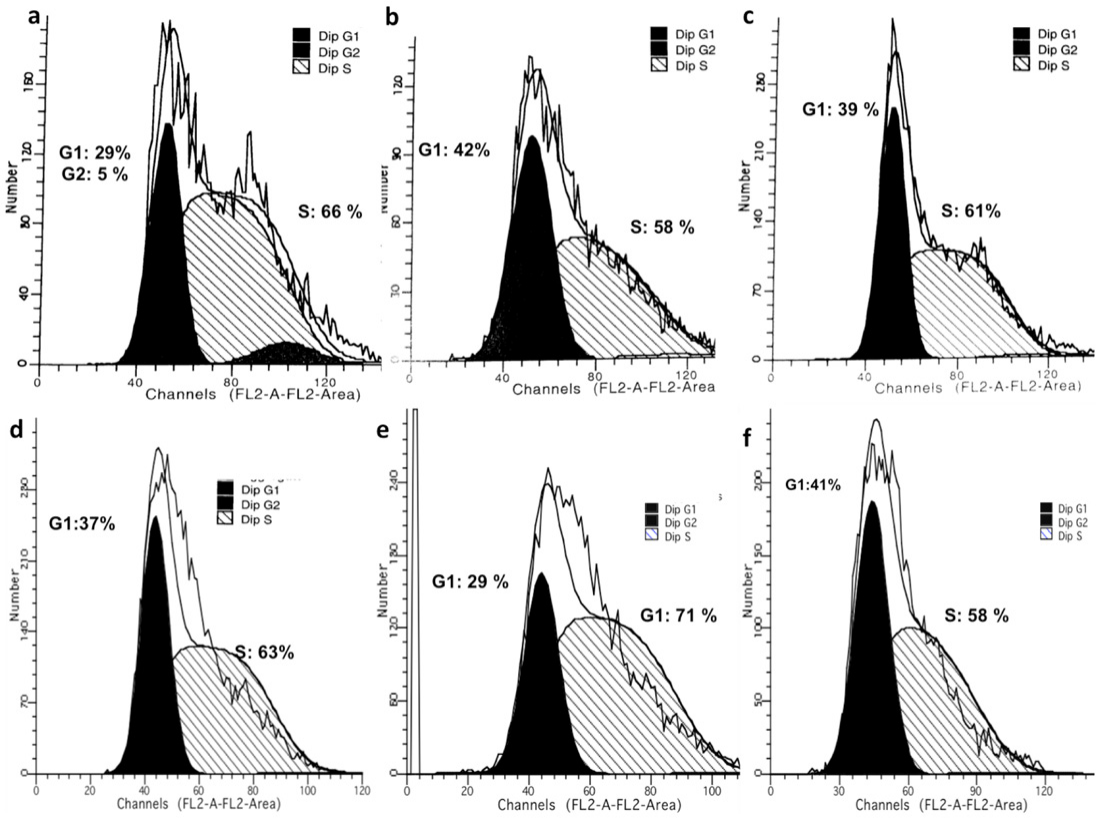
Analysis of cell cycle phase for non-treated BCCs by flow cytometry on: TCP at a) day 1 and d) day 7, random fibrous scaffolds at b) day 1 and e) day 7, and aligned fibrous scaffolds at c) day 1 and f) day 7.

On TCP for non-treated cells at day 1, 29% of the cells were in G0/G1 phase, 66% of the cells were in S phase, and 5% of the cells were in G2 phase (Fig. 9A). By day 7, 37% of the cells were in G0/G1 phase, 63% of the cells were in S phase and none of the cells cycled to G2 phase (Fig. 9D). Thus, more than 60% of non-treated BCCs were in S phase of the cell cycle for the 7 day culture period.

Non-treated BCCs on random scaffolds at day 1, showed that 42% were in G0/G1 phase, while 58% of the cells were in S phase, and none of the cells cycled to G2 phase (Fig. 9B). By day 7, 29% of the cells were in G0/G1 phase and 71% of the cells transitioned to the S phase while none of the cells cycled to G2 phase (Fig. 9E). Thus comparing day 1 to day 7, cells were more evenly distributed between G0/G1 phase and S phase at day 1, and by day 7 more cells were in the S phase than G0/G1 phase.

On aligned fibrous scaffolds at day 1, 39% of the non-treated cells were in G0/G1 phase, 61% of the cells were in S phase, and none of the cells cycled to G2 phase (Fig. 9C). By day 7, 42% of the cells were in G0/G1 phase, 58% of the cells were in S phase and none of the cells were in G2 phase (Fig. 9F). Thus at day 1, more cells were in the in the S phase than G0/G1 phase. However by day 7, cells were more evenly distributed in G0/G1 phase and in the S phase.

### Immunocytochemistry

Little to no cyclin D1 was expressed for non-treated BCCs on TCP (Fig. 5C). On fibrous scaffolds, non-treated BCCs stained for cyclin D1 during the 7-day culture period (Fig. 5A). Treated BCCs expressed cyclin D1 on TCP and fibrous scaffolds during the 7-day culture period (Fig. 5B). Similar to TCP, non-treated cells on random and aligned fibers stained for Bax at all time points unlike treated cells which did not express/had a weak stain for Bax at day 7 on random fiber scaffolds (S3 Fig. 1a-c). Both non-treated and treated cells expressed Bcl2 on all scaffolds at all time points, unlike both cell types on TCP at all time points (S3 Fig. 2a-c). Non-treated cells expressed Oct-4 on all scaffolds at all time points. Treated cells expressed Oct4 on random fibers at all time points. On aligned fibers, treated cells expressed Oct4 at day 1 but at day 7, the immunostain was weak/diminished for Oct4 (S3 Fig. 3a-c). Sox2 was highly expressed by non-treated cells and treated cells on all scaffolds at all time points (S3 Fig. 4a-c).

## Discussion

Breast cancer is one of the most common cancers diagnosed in women worldwide, and the second main cause of female mortality and morbidity in the western world [61]. Recurrences after decades of remission are a particular problem in breast cancer. This is mainly due to a subset of cells within the tumor microenvironment, which can remain in a non-dividing state of tumor dormancy. Patel et al. and others have previously reported that these cells, which represent cancer stem cells, exist in a quiescent state and can be resistant to traditional chemotherapeutic agents, which are generally designed to kill proliferating cells [4–7, 62, 63]. The biology of dormant cells is poorly understood. Tumor cells can persist either by completely withdrawing from the cell cycle or by continuing to proliferate at a slow rate. Moreover, dormant tumor cells share stem cell-like characteristics that may be responsible for their long half-lives and their resistance to standard chemotherapy[4]. Therefore, understanding the biology of tumor cell dormancy microenvironment may help develop innovative-targeted therapies to control or eliminate these resistant tumor cells. In *in vitro* studies, it is important to mimic the breast cancer cell microenvironment, which is known to be a 3-D structure consisting of neighboring cells, ECM, and blood vessels [64, 65]. Within this ECM, as cells form a 3-D structure, they rely on this microenvironment to maintain the ability to communicate and proliferate. Changes of the microenvironment can be fundamental to tumorigenesis, therefore the cellular behavior of cancer cells growing in conventional 2-D culture may be different from those *in vivo* in terms of proliferation, cellular signal transduction, and response to drugs [22, 25, 27, 29, 31, 33, 66, 67]. Thus, 3-D cultures may be used to study the mechanisms and effects by which these variations regulate cancer cell signaling [68, 69]. Recent research has indicated that side population cells resist chemotherapy and contain the cancer stem cells [21, 23, 24, 26, 28, 30, 32, 70]. Thus, in this study, to assess carboplatin sensitivity and isolate the dormant breast cancer cells, we exposed different breast cancer cell lines, T47D and MDA-MB-231 cells, to carboplatin concentrations ranging from 0 to 50 μg /ml for 72 hours and measured cytotoxicity to generate survival curves. All cell lines displayed sensitivity to carboplatin at 2-days and 3-days post treatments with the aggressive MDA-MB-231 cells having the least amount of cell death at the highest dosage for both 2- and 3-days post treatments. Additionally, carboplatin optimization for MDA-MB-231 cells indicated that even at a maximum carboplatin concentration of 220 μg /ml, these treated cells maintained higher percent viability of 80% as compared to 20% percent viability for the non-treated cells. Thus these treated BCCs were considered the most resistant side population. Carboplatin cell death can occur through an apoptotic pathway. Consequently, inhibition of this pathway by oncogenes genes such as Bcl-2 can lead to drug resistance [71]. The bcl-2 oncogene has been shown to have an anti-apoptotic function and may play a role in tumorigenesis by raising the threshold for apoptosis [58]. It has been well established that decreased levels of Bax are correlated to increased levels of Bcl-2. High expression levels of the BCL2 gene have been associated with drug resistance of the cancer cells [58, 60] Additionally, alongside being chemoresistant, these BCCs have been recognized to express genes linked to pluripotency [11, 15, 16]. Overall results by western analysis showed increased Bcl-2, Oct-4 and Sox-2 expression with increased carboplatin concentration which demonstrates that these resistant breast cancer cells have characteristics of cancer stem cell behavior [5–7]. On tissue culture polystyrene, carboplatin treatment of BCCs will kill the fast dividing proliferative cells and BCSCs that have differentiated into proliferative progenitors [4]. BCSCs have already been characterized to be quiescent (G0–G1 cell cycle arrest) without the effect of carboplatin [1, 4]. Moreover, CD44/CD24 has been widely used to isolate BCSCs [11]. Our results showed that the treated BCC population of cells was highly enriched with cells expressing CD44/CD24 markers. Thus along with the western results, these results indicated that by treating the heterogeneous BCC population of cells, we are selecting the resistant and breast cancer stem-like cells to seed on the scaffolds. From our results, MDA-MB-231 cells, which are generally known to be aggressive, withstood high carboplatin dosages without complete death and were selected to be cultured on the fibrous scaffolds to assess their proliferation, viability and cell cycle.

Using the electrospinning method, both random and aligned PCL fibrous scaffolds with uniform fiber morphology were fabricated for 3-D culture. The fibrous constructs produced by the electrospinning process have a high surface area-to-volume ratio, which can provide more surface for cell attachment as compared to TCP. We were able to fabricate random and aligned fibers with micron-sized fiber diameters. In tissue engineering, previous and recent studies have suggested a need for nanofibers for tissue formation and vascularization [72, 73]. However, the micron-sized fiber dimension more closely mimics the collagen fibers, which are bundled collagen fibrils, found in the native ECM. Microfiber scaffolds, in contrast to nanofiber scaffolds, have larger interfiber spacing [72, 74], which can better support cell infiltration and may promote an *in vivo*-like metastatic phenotype in terms of tumor morphogenesis (formation of spheroids), and migratory behavior related to the high invasiveness of MDA-MB-231cells [37]. Whereas, cells cultured on scaffolds with aligned fibers recognize the underlying geometry and thereby align themselves along the long axis of the fibers [37, 54, 75]. This indicates that aligned microfibers provide contact guidance, as was observed in our studies. Although there was a reduction in interfiber spacing for aligned as compared to random fiber scaffolds, cells on aligned scaffolds were still observed within the fibers. The modulus of elasticity for our random and aligned fibers was 4.4 MPa and 5.1 MPa, respectively, which is higher than epithelial basement membrane (~0.5MPa) [76] but only slightly higher than collagen fibrils (~2 MPa) [77]. However, the elastic modulus of the 2-D control, tissue culture polystyrene, is 2.5 GPa [78], which is three to four orders of magnitude higher than the PCL electrospun fibers. The stiffness of the extracellular matrix differs between tissues and it is altered in tumors. Results by Zahir et al. showed that the elastic modulus of the ECM has an effect on mammary epithelial cell morphogenesis that correlates with changes in actin organization and anti-apoptotic behavior [79, 80].

Non-treated and treated MDA-MB-231 cells cultured on the 3-D scaffold showed changes in cell morphology, adherence and viability. Visually analyzing the sub-volumes of cells in the 3-D-reconstructed Z-stack images showed that cells were able to penetrate and reside within the fibrous scaffolds. This is reminiscent of local metastatic breast cancer cells, which have been shown to migrate in direct contact along stromal collagen fibers[37, 81]. In agreement with this observation, our results showed that non-treated MDA-MB-231 cells on random scaffolds have a more round shape at day 1 and became more spread or spindle-like in shape at day 7. Unlike cells on fibrous scaffolds, cells seeded on TCP surface displayed confluency by day 7. Cell morphology is very important in early detection of the metastatic disease, and can be highly influenced by the tumor microenvironment which determines phenotype, gene expression, survival or apoptosis [80]. Both non-treated and treated MDA-MB-231 cells were within the random and aligned fibrous scaffolds by day 7. These findings suggest that the cancer cells may penetrate into the fibrous scaffolds and may reorient themselves in order to migrate through the fibrous structure. Several studies have shown that there is greater deformability of the cytoskeleton and nucleoskeleton in less differentiated stem cells, as compared to a decrease in deformability during differentiation to mature cells [82–85]. Moreover, increased tumor cell deformability has been correlated with increased metastatic potential [85–89]. Migration of cancer cells along fibers of remodeled ECM produced by prior invading cell(s) can result in distinctive cell alignment configurations. This form of invasion by sequences of breast cancer tumor cells linked together by cell–cell contacts has been shown to be an efficient penetration mechanism that can be correlated with high metastatic capacity and poor prognosis [90]. At day 1 of culture, some treated BCCs on random fibrous scaffolds, displayed attachment along the scaffold fibers, and switched to a more round-like shape at day 7. Treated BCCs on aligned fibrous scaffolds showed a more pronounced change in morphology where elongated actin filaments enveloped the scaffold fibers at day 1. By day 7, Oct 4 expression appeared weaker. Patel et al. reported a hierarchy of BCCs with the most immature subset (Oct4^hi^/CD44^hi/med^/CD24^-/+^) demonstrating chemoresistance, dormancy, and stem cell properties [4]. Moreover, gene analysis revealed that these BCCs cell subtype (with CD44^+^/CD24^−^/ claudin-low expression) also possessed higher flexibility that allowed them to change shape to pass through micro-barriers [4, 5, 91].

To further confirm whether the fibrous scaffolds can support the growth and viability of BCCs, we examined MDA-MB-231 cells in the 3-D fibrous scaffold environment. Results showed significantly different growth patterns between BCCs on 2-D surfaces as compared to 3-D fibrous scaffolds. Remarkably, the proliferative capacity of the usually aggressive MDA-MB-231 cells decreased on scaffolds relative to TCP inferring that these cells transitioned to a less aggressive phenotype when in contact with the fibrous scaffolds. Treated cells did not show any significant difference in growth between fibrous scaffolds and TCP suggesting that the growth of the treated cells was not affected by the 3-D scaffolds.

Expression of cyclin D1, a G1/S transition protein has been shown in several types of cancers [92]. In breast cancer, cyclin D1 is known to act as an oncogene, and its main role has been in the regulation of proliferation [92–94]. On fibrous scaffolds, non-treated cells expressed cyclin D1 during the 7-day culture period, unlike on TCP where little to no cyclin D1 was expressed. Treated BCCs expressed cyclin D1 on TCP and fibrous scaffolds during the 7-day culture period suggesting that these cells were perhaps approaching initiation of DNA synthesis or progressing through the remaining phases of the cell cycle. In line with the cyclin D1 expression findings, cell cycle analysis demonstrated that most of the aggressive MDA-MB-231 cells were either in the G0/G1 phase or arrested in the S phase during the 7-day culture period on fibrous scaffolds, unlike on TCP where G2 phase arrest occurred. These findings suggest that perhaps these cells have gone through early G1 cell cycle arrest where the protein was synthesized rapidly and accumulated steadily [95, 96]. These findings correlated with the previously obtained proliferation and viability results suggesting that the fibrous scaffolds did in fact affect the behavior of the different subsets of BCCs wherein aggressive cells display dormant characteristics on the scaffolds.

## Conclusion

In summary, within the tumor microenvironment, cancer stem cells (CSCs) are known to be tumor-initiating cells, major contributors of tumor growth, metastasis and recurrence. In the present work, we have fabricated 3-D fibrous scaffolds using the electrospinning process to evaluate how dormant and aggressive BCCs respond to this 3-D system. Proliferation, viability and cell cycle analysis indicated that 3-D culture prompted the aggressive BCCs to adopt a dormant phenotype, while the treated cancer cells retained their phenotype. This work provides a better understanding of how 3-D culture conditions affects BCC behavior. Findings also can lead to the design of novel biomaterials for the enrichment of CSCs as *in vitro* models for drug screening specifically targeting CSCs.

## Supporting Information

S1 Fig Densitometric bands of Bax, Bcl2, Oct4, and Sox2 expression of BCC treated with increasing dosage of carboplatin. Bands have been normalized to beta actin. A) Apoptosis related proteins for MDA: a) Bax and b) bcl2 and T47D: e) Bax and f) Bcl2. B) Self-renewal related proteins for MDA: c) Oct4 and d) Sox2 and T47D: g) Oct4 and h) Sox2
(TIF)

S2 Fig Representative SEM images of BCCs on fibrous scaffolds after 7 days of culture. a) Attachment sites indicated with arrows and b) BCCs within fibers. Scale bars are 2 μm. c) Cell-cell and cell-fiber interaction and d) cell-cell interaction on fibrous scaffolds. The arrows depict the cell body orientation along the fibers and the arrowheads show the fibers. Scale bars are 100 μm.
(TIFF)

S3 Fig Confocal fluorescent microscope images of expression Bax, Bcl2, Oct4, and Sox2 of MDA-MB-231 BCCs on the PCL random and aligned fibrous scaffolds and TCP control. Blue indicates nuclei (DAPI); green indicates F-actin (Alexa 488) and red is for anti-protein of interest. (Bax, Bcl2, Oct4, and Sox2). **S3.1 Expression of Bax** A) Non-treated BCCs on random scaffolds (a through d at day 1; e through h at day 7) and aligned scaffolds (i through l at day 1; m through p at day 7). B) Treated BCCs on random scaffolds (a through d at day 1; e through h at day 7) and aligned scaffolds (i through l at day 1; m through p at day 7). C) Non-treated BCCs (a through d at day 1; e through h at day 7) and treated BCCs (i through l at day 1; m through p at day 7) on TCP. **S3.2 Expression of Bcl2** A) Non-treated BCCs on random scaffolds (a through d at day 1; e through h at day 7) and aligned scaffolds (i through l at day 1; m through p at day 7). B) Treated BCCs on random scaffolds (a through d at day 1; e through h at day 7) and aligned scaffolds (i through l at day 1; m through p at day 7). C) Non-treated BCCs (a through d at day 1; e through h at day 7) and treated BCCs (i through l at day 1; m through p at day 7) on TCP. **S3.3 Expression of Oct4** A) Non-treated BCCs on random scaffolds (a through d at day 1; e through h at day 7) and aligned scaffolds (i through l at day 1; m through p at day 7). B) Treated BCCs on random scaffolds (a through d at day 1; e through h at day 7) and aligned scaffolds (i through l at day 1; m through p at day 7). C) Non-treated BCCs (a through d at day 1; e through h at day 7) and treated BCCs (i through l at day 1; m through p at day 7) on TCP. **S3.4 Expression of Sox2** A) Non-treated BCCs on random scaffolds (a through d at day 1; e through h at day 7) and aligned scaffolds (i through l at day 1; m through p at day 7). B) Treated BCCs on random scaffolds (a through d at day 1; e through h at day 7) and aligned scaffolds (i through l at day 1; m through p at day 7). C) Non-treated BCCs (a through d at day 1; e through h at day 7) and treated BCCs (i through l at day 1; m through p at day 7) on TCP. All scale bars are 50 μm. 100x objective.
(TIFF)
