# Supplementary figures and images for: Investigating Breast Cancer Cell Behavior Using Tissue Engineering Scaffolds
Source: PLoS One. 2015 Apr 2;10(4):e0118724. doi: 10.1371/journal.pone.0118724 (PMC4383476; doi:10.1371/journal.pone.0118724)

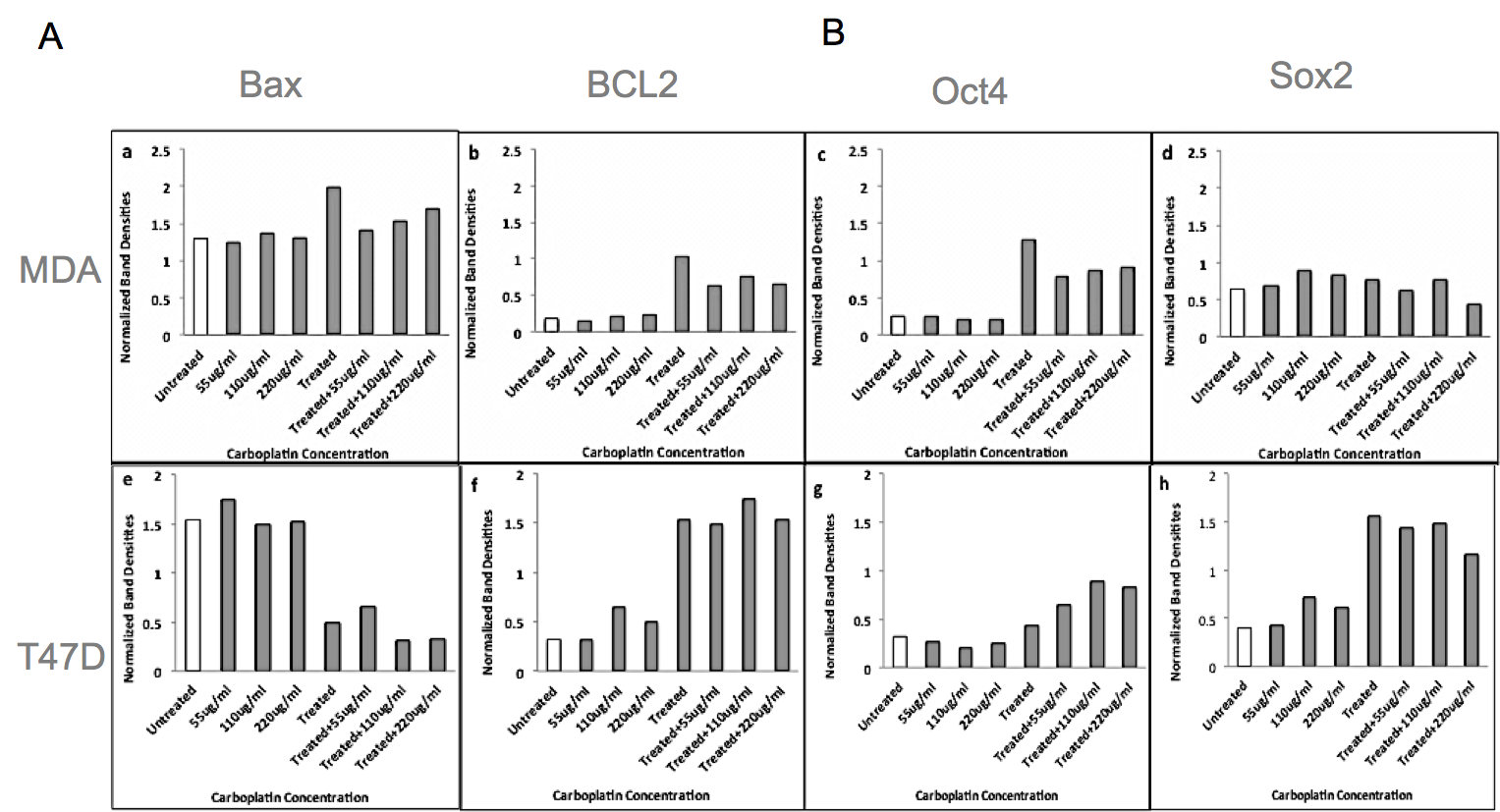

Supplement: S1 Fig — Bands have been normalized to beta actin. A) Apoptosis related proteins for MDA: a) Bax and b) bcl2 and T47D: e) Bax and f) Bcl2. B) Self-renewal related proteins for MDA: c) Oct4 and d) Sox2 and T47D: g) Oct4 and h) Sox2 (TIF) [file pone.0118724.s001.tif]

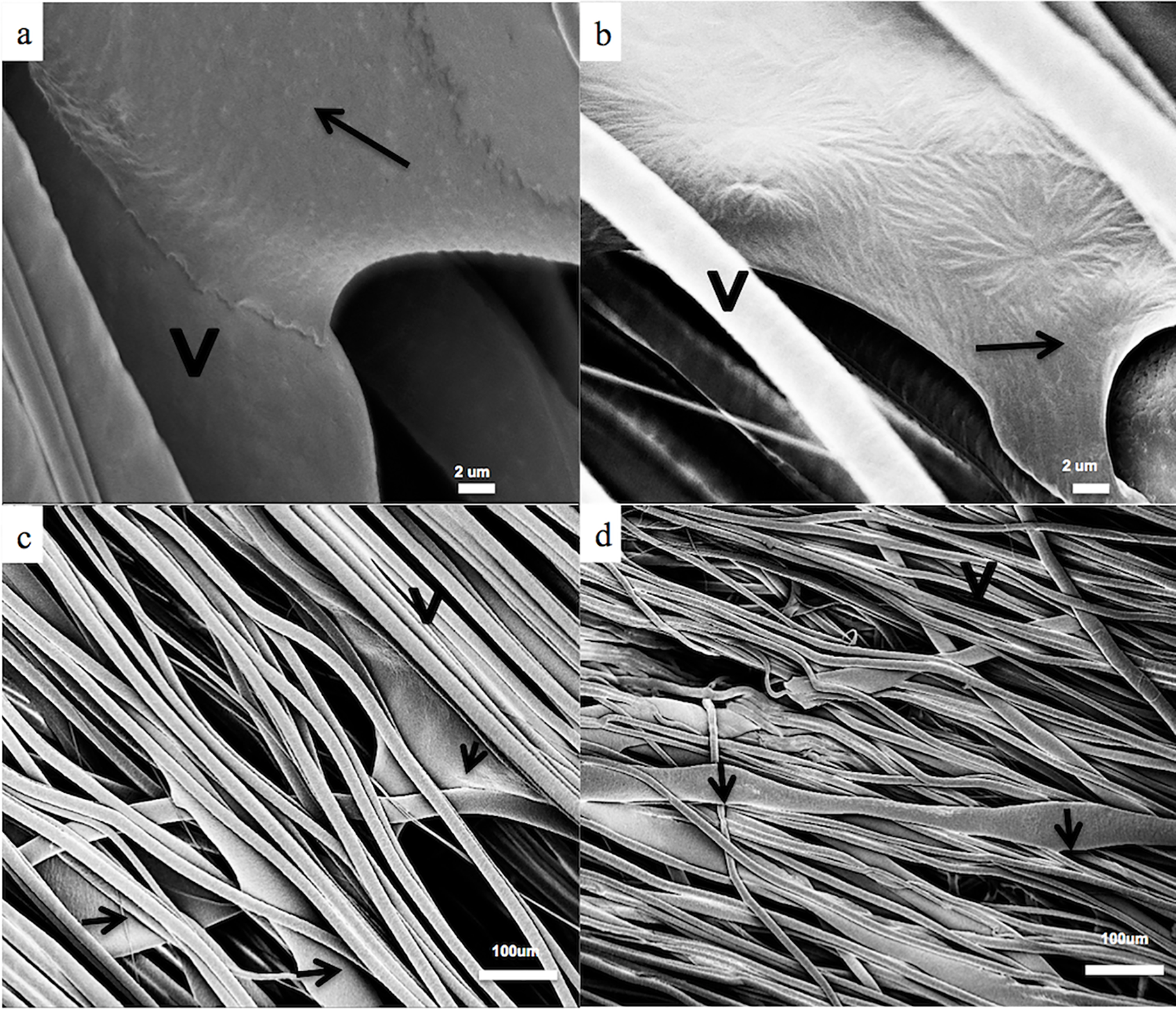

Supplement: S2 Fig — a) Attachment sites indicated with arrows and b) BCCs within fibers. Scale bars are 2 μm. c) Cell-cell and cell-fiber interaction and d) cell-cell interaction on fibrous scaffolds. The arrows depict the cell body orientation along the fibers and the arrowheads show the fibers. Scale bars are 100 μm. (TIFF) [file pone.0118724.s002.tiff]

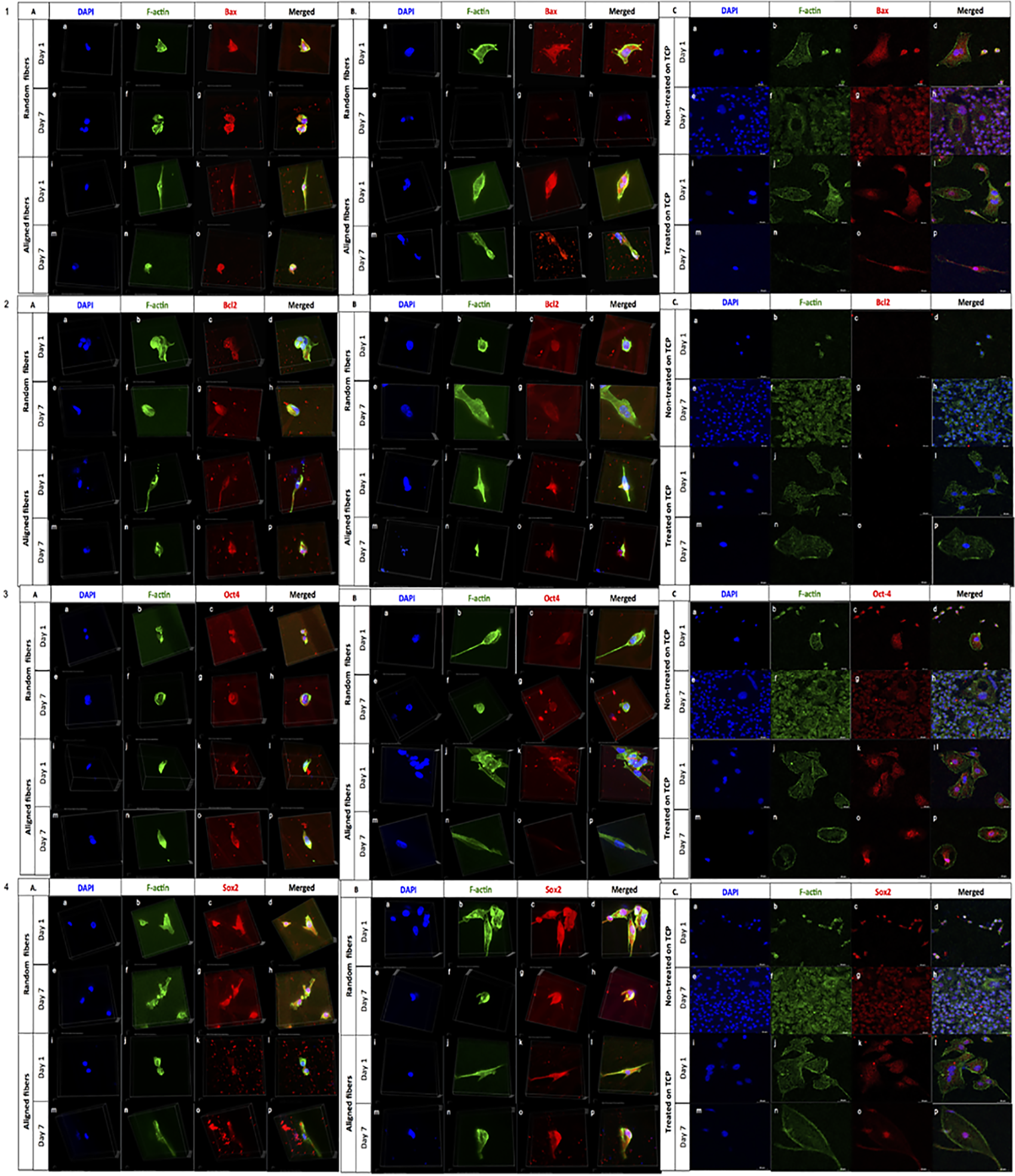

Supplement: S3 Fig — Blue indicates nuclei (DAPI); green indicates F-actin (Alexa 488) and red is for anti-protein of interest. (Bax, Bcl2, Oct4, and Sox2). S3.1 Expression of Bax A) Non-treated BCCs on random scaffolds (a through d at day 1; e through h at day 7) and aligned scaffolds (i through l at day 1; m through p at day 7). B) Treated BCCs on random scaffolds (a through d at day 1; e through h at day 7) and aligned scaffolds (i through l at day 1; m through p at day 7). C) Non-treated BCCs (a through d at day 1; e through h at day 7) and treated BCCs (i through l at day 1; m through p at day 7) on TCP. S3.2 Expression of Bcl2 A) Non-treated BCCs on random scaffolds (a through d at day 1; e through h at day 7) and aligned scaffolds (i through l at day 1; m through p at day 7). B) Treated BCCs on random scaffolds (a through d at day 1; e through h at day 7) and aligned scaffolds (i through l at day 1; m through p at day 7). C) Non-treated BCCs (a through d at day 1; e through h at day 7) and treated BCCs (i through l at day 1; m through p at day 7) on TCP. S3.3 Expression of Oct4 A) Non-treated BCCs on random scaffolds (a through d at day 1; e through h at day 7) and aligned scaffolds (i through l at day 1; m through p at day 7). B) Treated BCCs on random scaffolds (a through d at day 1; e through h at day 7) and aligned scaffolds (i through l at day 1; m through p at day 7). C) Non-treated BCCs (a through d at day 1; e through h at day 7) and treated BCCs (i through l at day 1; m through p at day 7) on TCP. S3.4 Expression of Sox2 A) Non-treated BCCs on random scaffolds (a through d at day 1; e through h at day 7) and aligned scaffolds (i through l at day 1; m through p at day 7). B) Treated BCCs on random scaffolds (a through d at day 1; e through h at day 7) and aligned scaffolds (i through l at day 1; m through p at day 7). C) Non-treated BCCs (a through d at day 1; e through h at day 7) and treated BCCs (i through l at day 1; m through p at day 7) on TCP. All scale bars are 50 [file pone.0118724.s003.tiff]
